# Liver Biopsy Technique for Analysis of Hepatic Content during Pregnancy and Early Lactation in Dairy Goats

**DOI:** 10.3390/vetsci11080384

**Published:** 2024-08-21

**Authors:** Aline Marangon de Oliveira, Anna Luiza Silva de Faria, Daiana Francisca Quirino, Érica Beatriz Schultz, Luciana Navajas Rennó, Marcelo Teixeira Rodrigues, Cristina Mattos Veloso

**Affiliations:** Animal Science Department, Universidade Federal de Viçosa, PH. Rolfs Avenue, Viçosa 36570-900, Brazil; aline.marangon@ufv.br (A.M.d.O.); anna.faria1@ufv.br (A.L.S.d.F.); daiana.villanova@ufv.br (D.F.Q.); erica.schultz@ufv.br (É.B.S.); lucianarenno@ufv.br (L.N.R.); mtrodrig@ufv.br (M.T.R.)

**Keywords:** glycogen, milk, pregnancy, production, triglycerides

## Abstract

**Simple Summary:**

Liver biopsy is a minimally invasive technique for liver evaluation, but its use is limited in dairy goats. Here, our main goal was to describe a liver biopsy method suitable for goat herds. The liver biopsy technique allows for the taking of liver samples to measure hepatic triglycerides and glycogen while preserving animal health and welfare. We evaluated the major factors that may cause changes in hepatic content in dairy goats, such as the peripartum period, number of fetuses, diet, supplementation, and milk production. The level of milk production and days relative to parturition influence hepatic triglyceride levels and glycogen content in dairy goats.

**Abstract:**

Biopsy techniques in dairy goats are currently limited. This study aimed to describe a liver biopsy technique in dairy goats and to evaluate liver triglyceride levels and glycogen content. Sixty-nine dairy goats in the final stage of pregnancy and early lactation period were selected. Fifty goats were selected randomly for hepatic biopsy (HB) according to gestational period and were characterized according to fetus number (single: *n* = 16, multiple: *n* = 34), supplementation with propylene glycol (diet: *n* = 23, diet+PG: *n* = 27), and milk production levels (high: 3.0 ± 0.4 L/day, n = 15; low: 1.4 ± 0.4 L/day, n = 26). Liver tissue samples were obtained through biopsy on days −30, −20, −15, −10, −5, and 15 days after calving. Hepatic triglyceride and glycogen were quantified. The results were analyzed using the F-test at a 5% significance level and a comparison of means using the Tukey test. The liver biopsies did not influence dry matter intake, body weight, or milk yield. Hepatic glycogen concentration was lower 15 days after calving than it was prior to calving, except on day −20. Goats that generated high levels of milk production had lower triglyceride levels than goats that generated low levels of milk production. The biopsy technique is a safe method for obtaining tissue and evaluating liver content in dairy goats. The milk production level and days relative to parturition influence the hepatic triglyceride and glycogen content in dairy goats.

## 1. Introduction

Liver biopsy is a minimally invasive technique for liver evaluation and is commonly used in production species, especially dairy cattle [1]. The biopsy, which involves cutting the liver with a simple trocar followed by aspiration, was initially developed for human liver sampling [2] and was later adapted for cattle [3]. For goats, the biopsy technique was described by Van den Top et al. [4] to evaluate the effects of dietary restriction on triglyceride and glycogen concentrations, even though the description was limited and instructional.

The liver is a crucial metabolic tissue for a successful transition between gestation and early lactation in ruminants. This transition is successful when there is a balance between the influx of fatty acids and lipid oxidation or export by the liver [5]. An imbalance between these factors increases fatty acid storage in the liver tissue through re-esterification to triacylglycerol [6]. The increases in hepatic triacylglycerol content may be accompanied by a lower hepatic glycogen content, indicating the occurrence of metabolic disorders [7].

Previous research has shown that certain factors increase the predisposition to metabolic disorders in dairy goats, such as the transition period [8,9], the number of fetuses [10], dairy production levels [11], and the diet [8]. These factors generally affect triglyceride influx into the liver. However, the relationship between these factors and hepatic triglyceride and glycogen content has not been researched in dairy goats.

Our first hypothesis is that the biopsy technique is a safe method of obtaining liver tissue in pregnant and lactating dairy goats without interfering with performance, dry matter intake, and milk production. Our second hypothesis is that the peripartum period, number of fetuses, milk production level, and supplementation with a gluconeogenic precursor can change liver triglyceride and glycogen content. Therefore, this study aimed to describe a safe liver biopsy technique for pregnant and lactating goats. In addition, this study analyzed the relationships between hepatic triglyceride and glycogen content and peripartum milk production levels, the number of fetuses, and diet supplementation with a gluconeogenic precursor.

## 2. Materials and Methods

The goats were selected from the herd of Universidade Federal de Viçosa (21°35′ S, 43°15′ W, 435 m above sea level, and a CWA-type climate, Köppen’s classification). Sixty-nine goats were selected, ranging from second to third lactations, with an average body weight (BW) of 68.5 ± 12.9 kg. The goats were of dairy breeds including Alpine (A, *n* = 26), Saanen (S, *n* = 25), and SXA crossbreeds (*n* = 18). The goats were kept in individual pens (1.75 m^2^) from the 120th day of gestation to the 15th day after calving. The animals had free access to food, water, and mineral salt. Fifty goats were randomly selected for hepatic biopsy (HB) according to gestational period. Nineteen goats did not undergo hepatic biopsy (NHB) and were kept in the same condition as the HB group (Table S1).

All the goats were fed a basal diet that consisted of corn silage and concentrate based on corn meal and soybean meal. The prepartum diet was formulated according to the pregnant goats’ nutritional requirements and averaged 55 kg of BW at 145 days of gestation, with a 1.5-kid prolificacy. The prepartum dietary composition was as follows: dry matter (DM), 445.2 g kg^−1^ natural matter; crude protein (CP), 125 g kg^−1^ DM; neutral detergent fiber (NDF), 334 g kg^−1^ DM; and metabolizable energy (ME) 2.9 Mcal kg^−1^ DM. The postpartum diet was formulated according to the lactating goat’s nutritional requirements and averaged 55 kg of BW, with 2.5 kg d^−1^ of milk yield for up to 30 days of lactation. The postpartum dietary composition was as follows: DM, 436.4 g kg^−1^ natural matter; CP, 188 g kg^−1^ DM; NDF, 351 g kg^−1^ DM; and ME, 2.8 Mcal kg^−1^ DM (Table S2). The requirements of both animal categories were calculated according to AFRC [12].

The goats were fed twice a day at 8:00 h and 15:00 h. The total diet was adjusted daily to ensure that DM represented 15% of the supplied feed. The daily control of dry matter intake (DMI) was achieved by weighing the amounts supplied to and refused by the goats. The DMI was recorded from day −30 to day 15 after calving. The BW was measured every 15 days and recorded from day −30 to day 30 after calving.

The goats were milked at 6:30 h and 14:30 h after calving. The milk yield was recorded until the 15th day post calving. During the experimental period, nine goats did not have their milk yield data recorded because one goat developed uterine prolapse, three goats had placenta retention, two goats had abortions, and three goats were diagnosed with clinical mastitis. Based on the milk data, the average milk yield per animal was calculated, and the goats were divided into a high-production group (3.0 ± 0.4 L/animal/day, *n* = 15) and a low-production group (1.4 ± 0.4 L/animal/day, *n* = 26) (Figure S1).

**Figure 1:**
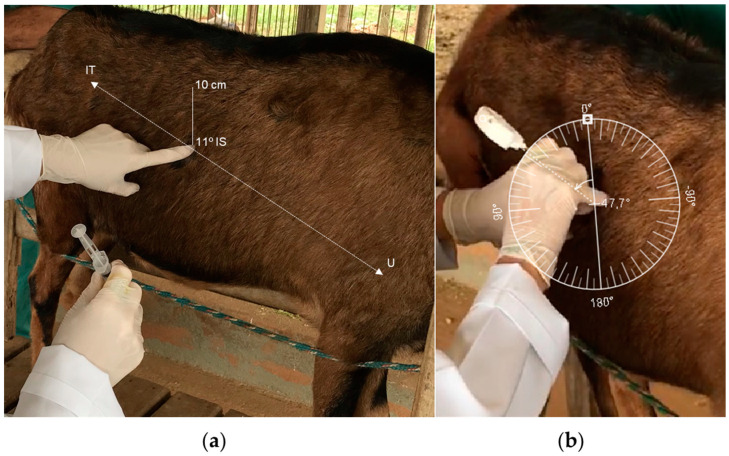
Liver biopsy in dairy goats. Animal was arranged in a standing position. (**a**) Side view of the incision site. An imaginary line between the iliac tuberosity (IT) and the mid-humeral region (U), approximately 10 cm below the transverse processes in the 12th or 11th intercostal space (IS). (**b**) Oblique cranioventral view showing the angle of the trocar during liver biopsy.

Liver tissue sampling was performed for each animal in the HB group throughout the experimental period. Collections were carried out on days −30 (*n* = 7), −20 (*n* = 6), −15 (*n* = 7), −10 (*n* = 10), −5 (*n* = 7), and 15 (*n* = 13) days after calving. The biopsy technique was performed after the goats were fed to guarantee the filling of the rumen. The goats were placed on a containment platform. The trichotomy was performed in the 12th or 11th intercostal space at the height of the iliac tuberosity approximately 10 cm below the vertebral transverse processes (Figure 1a), and the skin was cleaned with 70% alcohol. Then, 10 mL of 1% lidocaine was applied to the incision site. The anesthetic was applied superficially and deeply into the muscle layer. A small skin incision was made with a scalpel (0.3 cm). After the incision, a cannula with a solid and retractable pointed trocar model PICKUP no. PJT1115 (11 GA × 15 cm, Delebio, Italy) was introduced at approximately 45° in the cranioventral direction for the liver (Figure 1b). The cannula with the trocar was introduced three to four centimeters into the liver parenchyma. The trocar was removed, and a syringe was attached to the cannula for tissue extraction. At this stage, a subtle backward and forward movement of the tip of the cannula was made in an oblique direction towards the distal portion of the humerus. The liver sample was deposited in a Petri dish to clean the clots. The liver samples were stored in liquid nitrogen until analysis. After the biopsy, intramuscular oxytetracycline at 0.2 g/kg of BW was administered, and healing ointment was applied to each animal’s incision site.

The extraction of the triglycerides from the liver tissue was carried out according to the method proposed by Folch et al. [13], and triglyceride levels were quantified using a colorimetric enzymatic assay (K117, Bioclin^®^, Belo Horizonte, Brazil) with an automatic biochemistry analyzer (model BS200E) from Mindray^®^ (Shenzhen, China), following the manufacturer’s recommendations. For the extraction and quantification of hepatic glycogen, a Thermo Fisher Scientific^®^ (Vantaa, Finland) spectrophotometer (model Multiskan FC) set at a wavelength of 420 nm was used according to the methodology proposed by Carroll et al. [14], with modifications for the microplate reading.

To evaluate the factors influencing liver content, liver triglyceride and glycogen content of goats belonging to the HB group were evaluated. The following factors were also evaluated: days to calving (−30 days, *n* = 7; −20 days, *n* = 6; −15 days, *n* = 7; −10 days, *n* = 10; −5 days, *n* = 7; and 15 days, *n* = 13); number of fetuses (single, *n* = 16; twin, *n* = 34); milk yield (high production, n = 15; low production, *n* = 26); and diet (diet, *n* = 27; diet with propylene glycol supplementation, diet+PG, *n* = 23). Propylene glycol supplementation was administered beginning at the 135th day of gestation, with 23 goats receiving a daily oral dose of propylene glycol (90 mL/animal/day) until the 15th day after calving. This supplement added 0.408 Mcal/day to the diet’s level of metabolizable energy (ME).

Data from the collection of prepartum liver samples were retrospectively adjusted from the time of calving for data analysis.

Evaluations of BW, DMI, and milk yield in goats in the HB group and NHB group were carried out using ANOVA and included the fixed effect of the day. The means were compared using the Tukey test.

A statistical analysis of the factors was carried out using the following mathematical model (1):

Y_ijkl_ = µ + MP_i_ + NF_j_ + S_k_ + T_l_ + ε_ijkl_ (1)

where Y is the concentration of hepatic content; µ is the overall mean; MP is the fixed effect of milk production with two levels (low and high production); NF is the fixed effect of the number of fetuses during pregnancy with two levels (single and twin); S is the fixed effect of the supply of propylene glycol with two levels (diet and diet+PG); T is the fixed effect of the time of collection to calving (in days) with six levels (−30, −20, −15, −10, −5, and 15); and Ɛ is the random error. These factors were evaluated using the F-test at a 5% significance level, and the means were compared using Tukey test when necessary.

The errors were tested for normality using the Shapiro–Wilk test. Significance was declared at *p* < 0.05. All statistical procedures were analyzed using R software version 4.2.3.

## 3. Results

**Figure 2:**
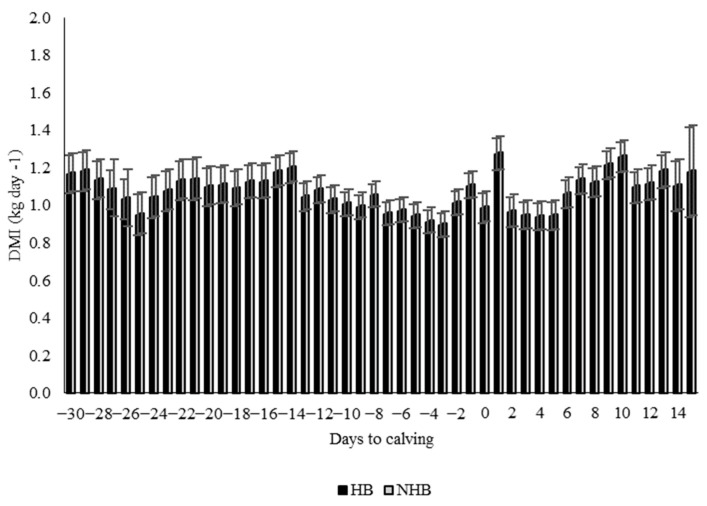
Dry matter intake (DMI) of dairy goats that underwent a hepatic biopsy (HB) or that did not undergo a hepatic biopsy (NHB); the time period of measurement was four weeks before calving to two weeks after calving.

Immediately after the liver biopsies, no goat presented with clinical complications. DMI was similar between the HB group (1.07 ± 0.01 kg day^−1^) and the NHB group (1.08 ± 0.02 kg day^−1^, *p* = 0.712, Figure 2).

**Figure 3:**
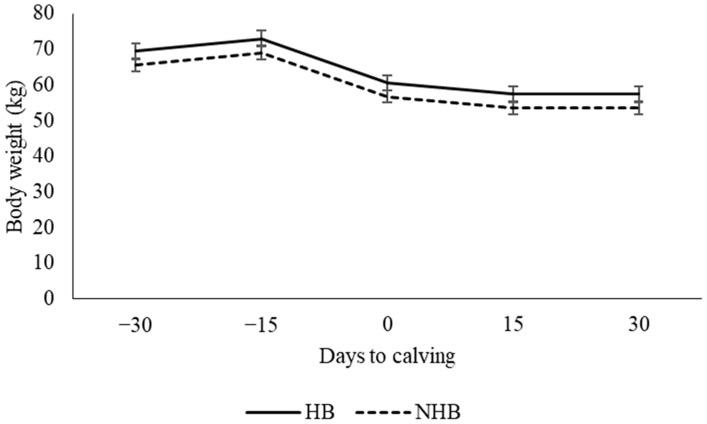
Body weight of dairy goats that underwent a hepatic biopsy (HB) or that did not undergo a hepatic biopsy (NHB); the time period of measurement was four weeks before calving to two weeks after calving.

The liver biopsies did not influence the BW (*p* = 0.4842, Figure 3). Milk yield was similar between the HB group (2.02 ± 0.14 L day^−1^) and the NHB group (2.31 ± 0.14 L day^−1^, *p* = 0.363). The liver sample tissue weight was 400 mg, allowing for an evaluation of hepatic triglyceride and glycogen content.

**Figure 4:**
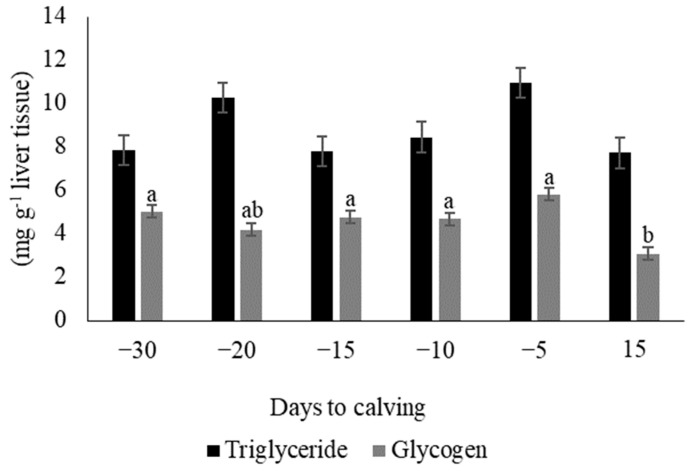
Hepatic triglyceride and glycogen content in dairy goats four weeks before calving to two weeks after calving. Different letters indicate statistical differences (*p* = 0.010) between days.

The hepatic glycogen content was lower (*p* = 0.01) 15 days (3.08 ± 1.5 mg g^−1^ of liver tissue) after calving than it was prepartum, except on day −20 (4.17 ± 1.5 mg g^−1^ of liver tissue, Figure 4). The hepatic triglyceride content was similar during prepartum and postpartum (*p* = 0.49, Figure 4).

**Figure 5:**
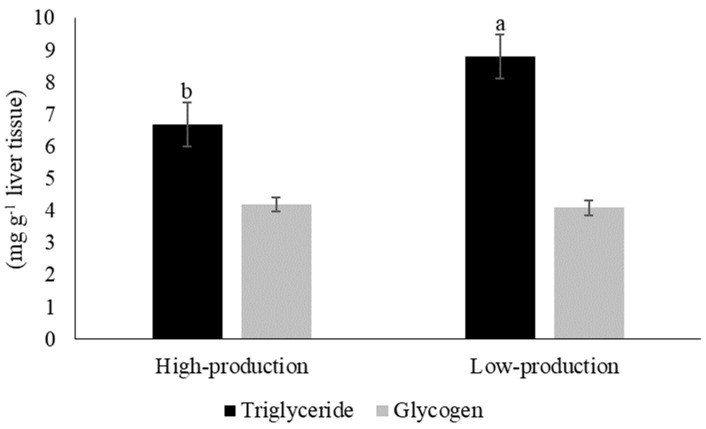
Hepatic triglyceride and glycogen content in dairy goats in the high-production (3.0 ± 0.4 L d^−1^) and low-production groups (1.4 ± 0.4 L d^−1^). Different letters indicate statistical differences (*p* = 0.042).

Goats that generated high levels of milk production (HP, 6.7 ± 1.6 mg g^−1^ of liver tissue) had a lower (*p* = 0.04) hepatic triglyceride content than goats that generated low levels of milk production (LP, 8.8 ± 2.2 mg g^−1^ of liver tissue). However, the hepatic glycogen content was similar between the high-production and low-production groups (*p* = 0.71, Figure 5).

The number of fetuses and propylene glycol supplementation did not influence hepatic triglyceride and glycogen content (*p* > 0.05, Table 1).

## 4. Discussion

Liver biopsy is used as a tool for evaluating disorders and diagnoses. The correct execution of the technique is essential for satisfactory tissue collection without compromising animal welfare and health. Our main goal was to describe a method that can be applied to goat herds, and used by veterinarians in the field. Liver evaluations allow researchers access to knowledge about metabolic diseases and their treatment. Among metabolic diseases, understanding lactation ketosis and pregnancy toxemia is essential to reduce economic losses in high-production herds.

Changes in liver levels of certain biomarkers are observed during the transition period in dairy cows [15]. In the final trimester of pregnancy, there is an increase in energy demand due to fetal development. However, as the fetus grows, rumen compression occurs, reducing the capacity for feed intake. Additionally, there is extensive hormonal regulation of glucose allocation to tissues, which prioritizes the pregnant uterus and the development of mammary glands [6]. As a physiological response, adipose tissue is mobilized to the liver, which changes its liver content [16]. However, our results did not show significant differences in hepatic triglyceride content on the days relative to calving. The reduction in postpartum hepatic glycogen content observed in our study might be associated with lactation. Similar results were observed by Van den Top et al. [4] in dairy goats with free access to feed.

Goats in the low-production group had a high level of fat infiltration in the liver. In dairy cows [16], a fat infiltration greater than 9.5% of liver tissue reduces milk yield. Higher fat infiltration in the liver promotes potential damage to hepatocytes. This damage reduces the liver’s capacity to convert propionate into glucose and transform ammonia into urea [7], consequently reducing the levels of the components necessary for milk synthesis. However, in dairy cows, a fat infiltration of less than 7.5% of liver tissue does not influence the animal’s performance [16]. Similarly, we observed a lower fat infiltration in goats in the high-production group in our study.

The number of fetuses in goats might result in higher liver triglyceride accumulation. The increased flow of lipids to the liver could be influenced by the higher nutritional requirements of the fetuses during the final stages of gestation [8]. However, our results did not show any differences in liver triglyceride accumulation between goats that had a single pregnancy and goats that had a twin pregnancy. Accordingly, Doré et al. [10] there is an increase in nutritional requirements when the number of fetuses is higher than three per pregnancy.

Propylene glycol supplementation might promote a decrease in lipid mobilization and an increase in glucose in tissues [17,18]. This supposedly occurs because propylene glycol reduces beta-oxidation in hepatocytes [19]. The supplementation in our study did not influence hepatic fat content. The diet provided to the animals was likely sufficient to maintain energy balance during the physiological changes required during the period of study, reducing lipid mobilization even without the addition of propylene glycol.

In dairy cows, the hepatic fat content has been extensively analyzed in nutritional and metabolic studies [7,16]. The increased hepatic triglyceride content is associated with health, milk production, and a reduction in reproductive performance [20,21,22]. These associations have encouraged further research into liver status in dairy cows. Despite the intensification of milk production in goats [23], this is the first report of the association between productive factors and liver content in pregnant and lactating goats.

## 5. Conclusions

Our results showed that the proposed biopsy technique is safe for obtaining liver samples from dairy goats during pregnancy and early lactation. The biopsy technique allows the adequate sampling of hepatic tissue and does not affect animal performance. The fetus number and propylene glycol supplementation did not influence the hepatic triglyceride and glycogen content. However, the triglyceride content was lower in dairy goats that generated high levels of milk production, and the glycogen content was reduced postpartum.

## Figures and Tables

**Table 1:** Hepatic triglyceride and glycogen content in dairy goats according to the number of fetuses and propylene glycol (PG) supplementation.

Variable	Triglycerides ^1^	Glycogen ^1^
Number of fetuses		
Single	7.9 ± 4.6	4.36 ± 1.8
Twin	9.1 ± 5.2	4.36 ± 2.1
*p*-value	0.615	0.669
Supplementation		
Diet	8.0 ± 6.0	4.4 ± 3.4
Diet+PG	9.3 ± 4.9	4.5 ± 1.9
*p*-value	0.422	0.919

^1^ mean ± deviation.

## Data Availability

The raw data supporting the conclusions of this article will be made available by the authors on request.

## Supplementary Materials

The following supporting information can be downloaded at: https://www.mdpi.com/article/10.3390/vetsci11080384/s1, Table S1. Dairy breeds, number of fetuses, diet and supply of dairy goats that underwent a hepatic biopsy (HB) or that did not undergo a hepatic biopsy (NHB) during peripartum. Table S2. Ingredients and diet composition of prepartum and postpartum for dairy goats according to AFRC requirements (1993). Figure S1. Milk yield of dairy goats that underwent a hepatic biopsy (HB) or that did not undergo a hepatic biopsy (NHB).
