# Supplementary material for: Liver Biopsy Technique for Analysis of Hepatic Content during Pregnancy and Early Lactation in Dairy Goats
Source: Vet Sci. 2024 Aug 21;11(8):384. doi: 10.3390/vetsci11080384 (PMC11359292; doi:10.3390/vetsci11080384)
Supplement: Supplementary file 1 [file vetsci-11-00384-s001.zip › Figure S1.pdf]

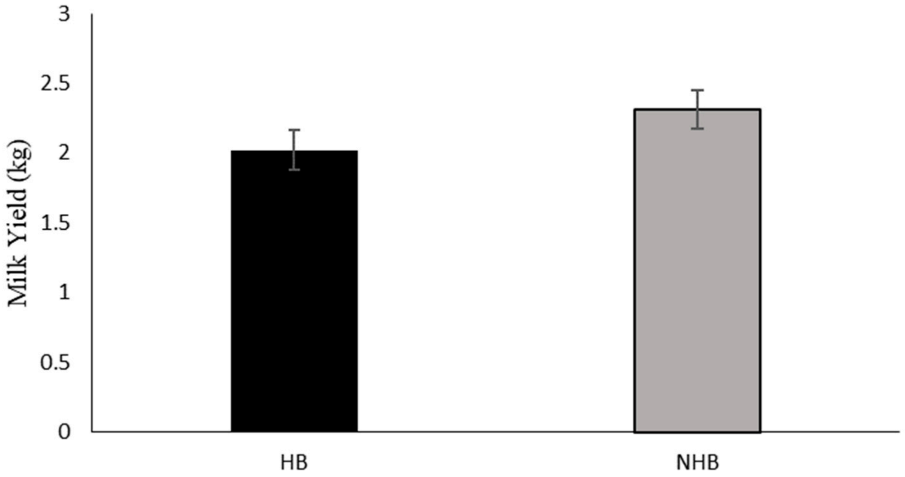

Figure S1. Milk yield of dairy goats that underwent a hepatic biopsy (HB) or that did not undergo a hepatic biopsy (NHB).
