# Supplementary material for: Liver Biopsy Technique for Analysis of Hepatic Content during Pregnancy and Early Lactation in Dairy Goats
Source: Vet Sci. 2024 Aug 21;11(8):384. doi: 10.3390/vetsci11080384 (PMC11359292; doi:10.3390/vetsci11080384)
Supplement: Supplementary file 1 [file vetsci-11-00384-s001.zip › Table S1.pdf]

Table S1. Dairy breed, number of fetuses, diet and supply of dairy goats that underwent a hepatic biopsy (HB) or that did not undergo a hepatic biopsy (NHB) during peripartum.

ID	Breed[1]	Number of fetuses	Diet+Supply	Group	Days to calving
5501	Saanen	Twins	Diet+PG	HB	-15
5564	SXA	Twins	Diet	HB	-5
5594	Saanen	Twins	Diet	HB	-10
5604	SXA	Twins	Diet+PG	HB	15
5612	SXA	Twins	Diet+PG	HB	-20
5625	Alpine	Twins	Diet	HB	-15
5639	Saanen	Twins	Diet	HB	-30
5662	Saanen	Twins	Diet	HB	-5
5666	Saanen	Twins	Diet	HB	15
5685	Saanen	Twins	Diet	HB	15
5688	Saanen	Twins	Diet+PG	HB	15
5695	SXA	Twins	Diet+PG	HB	-15
5696	SXA	Singleton	Diet	HB	-30
5701	Saanen	Singleton	Diet+PG	HB	15
5706	SXA	Singleton	Diet+PG	HB	-20
5712	Alpine	Twins	Diet	HB	15
5734	Saanen	Twins	Diet	HB	15
5741	Saanen	Twins	Diet+PG	HB	-10
5750	Alpine	Twins	Diet+PG	HB	-5
5775	Alpine	Singleton	Diet	HB	-5
5791	Saanen	Twins	Diet	HB	-15
5796	Alpine	Twins	Diet+PG	HB	15
5800	Saanen	Twins	Diet	HB	-5
5801	Alpine	Twins	Diet+PG	HB	-15
5807	Saanen	Twins	Diet+PG	HB	15
5810	SXA	Twins	Diet+PG	HB	-10
5811	SXA	Singleton	Diet+PG	HB	-10
5813	Saanen	Singleton	Diet	HB	-15
5823	SXA	Singleton	Diet+PG	HB	15
5826	Alpine	Twins	Diet	HB	-10
5830	SXA	Twins	Diet	HB	-10
5837	Alpine	Twins	Diet+PG	HB	-10
5838	SXA	Twins	Diet+PG	HB	-10
5849	SXA	Twins	Diet	HB	15
5858	Alpine	Twins	Diet	HB	-5
5862	Saanen	Twins	Diet+PG	HB	-15
5864	Alpine	Singleton	Diet+PG	HB	-10
5877	Alpine	Singleton	Diet	HB	-5
5882	Alpine	Twins	Diet+PG	HB	-30
5887	Alpine	Singleton	Diet+PG	HB	15
5889	Alpine	Twins	Diet+PG	HB	15
5932	Saanen	Twins	Diet	HB	-10
5953	Alpine	Singleton	Diet	HB	-20
5965	SXA	Twins	Diet	HB	-30

5973	Alpine	Singleton	Diet	HB	-30
5980	Alpine	Singleton	Diet	HB	-30
6015	Saanen	Singleton	Diet	HB	-20
6026	SXA	Singleton	Diet	HB	-20
6031	Alpine	Singleton	Diet	HB	-30
5615	Alpine	Twins	Diet	NHB	NA
5649	Alpine	Singleton	Diet	NHB	NA
5659	SXA	Twins	Diet	NHB	NA
5686	Saanen	Twins	Diet	NHB	NA
5719	Saanen	Twins	Diet+PG	NHB	NA
5724	Saanen	Twins	Diet	NHB	NA
5782	SXA	Twins	Diet	NHB	NA
5842	Saanen	Twins	Diet	NHB	NA
5920	Alpine	Twins	Diet	NHB	NA
5921	Alpine	Twins	Diet	NHB	NA
5946	Saanen	Twins	Diet	NHB	NA
5947	Alpine	Singleton	Diet	NHB	NA
5964	SXA	Singleton	Diet	NHB	NA
5966	SXA	Twins	Diet	NHB	NA
5970	Saanen	Singleton	Diet	NHB	NA
5975	Alpine	Twins	Diet	NHB	NA
5983	Saanen	Twins	Diet	NHB	NA
6001	Saanen	Twins	Diet	NHB	NA
6048	Alpine	Singleton	Diet	NHB	NA

[1]SXA Saanen x Alpina.
