# Supplementary material for: Liver Biopsy Technique for Analysis of Hepatic Content during Pregnancy and Early Lactation in Dairy Goats
Source: Vet Sci. 2024 Aug 21;11(8):384. doi: 10.3390/vetsci11080384 (PMC11359292; doi:10.3390/vetsci11080384)
Supplement: Supplementary file 1 [file vetsci-11-00384-s001.zip › Table S2.pdf]

Table S2. Ingredients and diet composition of prepartum and postpartum for dairy goats according to AFRC requirements (1993)

Item	Prepartum	Postpartum
Ingredients[1]		
Corn silage	494.4	528.4
Corn	362.5	197.8
Soybean meal	112.6	255.1
Calcium carbonate	10.5	8.3
Bicalcium phosphate	10.4	0.5
Sodium bicarbonate	-	10.0
Diet composition[2]		
DM[3]	445.2	436.4
CP[4]	125	188
NDF[5]	334	351
ME[6]	2.9	2.8
ME+PG[7]	3.3	3.2

[1] g kg^{-1}.
[2] Approximated values.
[3]DM: dry matter (g kg^{-1} natural matter).
[4] CP: crude protein (g kg^{-1} DM).
[5] NDF: neutral detergent fiber (g kg^{-1} DM).
[6]ME: metabolizable energy (Mcal kg^{-1} DM).
[7]ME + PG: total metabolizable energy (Mcal kg^{-1} DM) considering the supply of propylene glycol (PG, 0.408 Mcal).
